# Supplementary material for: Impact of Preprocedural Collateral Status on Hemorrhagic Transformation and Outcomes After Endovascular Thrombectomy in Acute Ischemic Stroke
Source: Diagnostics (Basel). 2025 Oct 25;15(21):2701. doi: 10.3390/diagnostics15212701 (PMC12607835; doi:10.3390/diagnostics15212701)
Supplement: Supplementary file 1 [file diagnostics-15-02701-s001.zip › diagnostics-3887904-supplementary.pdf]

Supplementary Tables S1–S2 (Sensitivity Analysis Excluding PTA/Stent)

These supplementary tables replicate the main analyses after removing patients who underwent rescue percutaneous transluminal angioplasty and/or stenting. Statistical tests and units follow the main tables.

Supplementary Table S1. Hemorrhagic transformation analysis (patients without PTA/stent; total n=68).

Groups: Non-HT (n=28) vs HT (n=40). Values are median (IQR) or n (%).

Variable	Total (n=68)	Hemorrhagic transformation		*p* value
		non-HT (n=28)	HT (n=40)	
**Age, median (IQR)**	70.5 (60.3 - 78)	70 (60.3 - 75.5)	72 (59.5 - 79)	0.386
**Gender, n (%)**				0.333
Female	29 (42.6%)	10 (35.7%)	19 (47.5%)	
Male	39 (57.4%)	18 (64.3%)	21 (52.5%)	
**BMI (kg/m^2), median (IQR)**	23.9 (21.4 - 26.5)	24.9 (21.4 - 27.2)	22.8 (21.1 - 26.2)	0.404
**Comorbidity, n (%)**				
Hypertension	38 (55.9%)	14 (50%)	24 (60%)	0.414
Diabetes Mellitus	23 (33.8%)	11 (39.3%)	12 (30%)	0.426
Dyslipidemia	48 (70.6%)	19 (67.9%)	29 (72.5%)	0.679
Atrial fibrillation	41 (60.3%)	17 (60.7%)	24 (60%)	0.953
Previous tPA	32 (47.1%)	19 (67.9%)	13 (32.5%)	0.004**
**Laboratory data, median (IQR)**				
Hb, g/dL	13.7 (12.1 - 15)	13.6 (12 - 14.9)	13.8 (12.1 - 15.2)	0.636
WBC, , cells/μL	7970 (6117.5 - 10217.5)	8595 (5965 - 11462.5)	7535 (6222.5 - 9587.5)	0.301
Platelet, ×10^3/μL	196 (160.8 - 246.5)	185.5 (165.8 - 242.5)	211 (157.8 - 252.3)	0.667
Cholesterol, mg/dL	150 (131.5 - 176.5)	146 (125.5 - 167.8)	155 (137 - 208.3)	0.049*

LDL, mg/dL	88 (71 - 114)	84.5 (66.3 - 102.8)	93 (75 - 130)	0.044*
**Repurfusion time, median (IQR)**				
Onset to repurfusion (hours)	5.9 (4.5 - 7.7)	5.5 (4.4 - 7.1)	6.3 (4.5 - 8.8)	0.217
Door to repurfusion (hours)	2.6 (2 - 3.2)	2.6 (2.2 - 3.5)	2.6 (1.9 - 3)	0.554
Puncture to repurfusion (hours)	0.7 (0.5 - 1.1)	0.8 (0.5 - 1.2)	0.6 (0.4 - 1.1)	0.533
**NIHSS, median (IQR)**				
ER NIHSS	17.5 (13 - 21)	17 (13 - 19)	19 (13 - 22.8)	0.158
Post-24h NIHSS	11 (6 - 16.3)	8 (4 - 13.8)	13.5 (7 - 18)	0.012*
NIHSS improved	5 (2.8 - 10.3)	7.5 (4.8 - 12)	5 (2 - 10)	0.091
Vessel side, n (%)				0.748
Left	38 (55.9%)	15 (53.6%)	23 (57.5%)	
Right	30 (44.1%)	13 (46.4%)	17 (42.5%)	
Vessel location, n (%)				1.000
MCA	53 (77.9%)	22 (78.6%)	31 (77.5%)	
ICA	5 (7.4%)	2 (7.1%)	3 (7.5%)	
Combined	10 (14.7%)	4 (14.3%)	6 (15%)	
**Pre-procedural CT, median (IQR)**				
CBF <30% of CTP	23.5 (4.5 - 43.8)	18 (0 - 46.8)	26.5 (6.8 - 43)	0.667
Tmax > 6sec of CTP	99 (71.8 - 131)	99 (75 - 138)	99.5 (67.3 - 130.3)	0.536
Tmax > 10sec of CTP	65 (21.8 - 81.8)	60 (30 - 90.5)	65 (17.5 - 80)	0.597
Hypoperfusion index ratio	0.6 (0.4 - 0.7)	0.5 (0.4 - 0.7)	0.6 (0.4 - 0.7)	0.831
Mismatch ration	2.9 (2.3 - 5.4)	2.5 (2.1 - 11.9)	3.1 (2.3 - 4.4)	0.913
Collateral score	4 (3 - 4)	4 (3.3 - 4.8)	4 (3 - 4)	0.068
Collateral score, n (%)				0.198
Poor	45 (66.2%)	21 (75%)	24 (60%)	
Good	23 (33.8%)	7 (25%)	16 (40%)	
NCCT-ASPECTS	9 (8 - 9)	9 (8 - 9)	8 (8 - 9)	0.006**
**Procedural, n (%)**				

mTICI				0.523
2B	17 (25%)	5 (17.9%)	12 (30%)	
2C	9 (13.2%)	4 (14.3%)	5 (12.5%)	
3	42 (61.8%)	19 (67.9%)	23 (57.5%)	
**Post-procedural DECT, median (IQR)**				
sNCCT-ASPECTS	8 (6 - 9)	9 (7.3 - 9)	7 (5 - 8)	<0.001**
VNC-ASPECTS	6 (5 - 8)	8 (6.3 - 9)	6 (4 - 7)	<0.001**
IOM-ASPECTS	6 (5.5 - 7.5)	5.5 (2 - 6.8)	6 (6 - 8)	0.188
**Post-procedural MRI**				
Ischemic volume (mm^3) by MRI, median (IQR)	3.6 (-20.3 - 28.9)	4.9 (-13.9 - 31.8)	3.6 (-23.9 - 25.9)	0.702
DWI-ASPECT, median (IQR)	5 (4 - 6)	6 (5 - 8)	5 (3.3 - 6)	0.002**
**Clinical Outcomes, n (%)**				
Neurosurgical intervention	3 (4.4%)	2 (7.1%)	1 (2.5%)	0.564
Inhospital length, median (IQR)	15 (9 - 19.8)	12 (8 - 15)	17 (14 - 24.3)	<0.001**
Favorable mRS at 3-month	30 (44.1%)	16 (57.1%)	14 (35%)	0.070
Excellent mRS at 3-month	16 (23.5%)	12 (42.9%)	4 (10%)	0.002**
3-month mortality	6 (8.8%)	2 (7.1%)	4 (10%)	1.000
NCCTDWI, median (IQR)	3 (2 - 4)	3 (1 - 4)	3 (2 - 5)	0.095
VNCDWI, median (IQR)	0 (0 - 2)	1 (0 - 2.8)	0 (0 - 1)	0.195
Ischemic volume trend, median (IQR)	21471 (7186.5 - 46810.5)	23013 (8127 - 77734.5)	21375 (5484 - 46549.5)	0.960

*Data are median (IQR) or n (%). Units: Age, years; BMI, kg/m^2; Hb, g/dL; WBC, cells/µL; Platelet, ×10^3/µL; Cholesterol/LDL, mg/dL; time intervals (onset-to-, door-to-, puncture-to-reperfusion), hours; in-hospital length, days; CTP volumes (CBF <30%, Tmax >6 s, Tmax >10 s), mL; MRI ischemic volume, mL. Scores are unitless unless otherwise noted: NIHSS (points), mRS, collateral score (0–5), ASPECTS (0–10), mTICI (2B–3). ASPECTS differences (NCCTDWI, VNCDWI) are in points; ischemic volume trend is in mL. Tests: Mann–Whitney U or Kruskal–Wallis for continuous variables; chi-square or Fisher (or Fisher–Freeman–Halton) for categorical variables. Significance: *p*<0.05; **p**<0.01.*

Supplementary Table S2. Hemorrhagic transformation × collateral status analysis (patients without PTA/stent; total n=68).

Groups: Non-HT (n=28), HT with good CS (n=24), HT with poor CS (n=16). Values are median (IQR) or n (%).

Variable	Total (n=68)	Hemorrhagic transformation & Collateral score			*p* value
		Non-HT (n=28)	HT & Good CS (n=24)	HT & Poor CS (n=16)	
**Age, median (IQR)**	70.5 (60.3 - 78)	70 (60.3 - 75.5)	72 (59.5 - 81.3)	72 (61.5 - 79)	0.677
**Gender, n (%)**					0.149
Female	29 (42.6%)	10 (35.7%)	14 (58.3%)	5 (31.3%)	
Male	39 (57.4%)	18 (64.3%)	10 (41.7%)	11 (68.8%)	
**BMI (kg/m^2), median (IQR)**	23.9 (21.4 - 26.5)	24.9 (21.4 - 27.2)	22.6 (20.2 - 25.5)	25.6 (22.3 - 28.7)	0.048*
**Comorbidity, n (%)**					
Hypertension	38 (55.9%)	14 (50%)	14 (58.3%)	10 (62.5%)	0.692
Diabetes Mellitus	23 (33.8%)	11 (39.3%)	6 (25%)	6 (37.5%)	0.521
Dyslipidemia	48 (70.6%)	19 (67.9%)	18 (75%)	11 (68.8%)	0.839
Atrial fibrillation	41 (60.3%)	17 (60.7%)	14 (58.3%)	10 (62.5%)	0.964
Previous tPA	32 (47.1%)	19 (67.9%)	6 (25%)	7 (43.8%)	0.008**
**Laboratory data, median (IQR)**					
Hb, g/dL	13.7 (12.1 - 15)	13.6 (12 - 14.9)	13.7 (12 - 15.4)	13.9 (13.1 - 15.1)	0.774
WBC, cells/μL	7970 (6117.5 - 10217.5)	8595 (5965 - 11462.5)	7330 (6380 - 9790)	7890 (5245 - 9375)	0.550
Platelet, ×10^3/μL	196 (160.8 - 246.5)	185.5 (165.8 - 242.5)	223.5 (165.3 - 285)	171.5 (142.5 - 221)	0.120
Cholesterol, mg/dL	150 (131.5 - 176.5)	146 (125.5 - 167.8)	158 (138 - 208)	141 (133 - 210)	0.135

LDL, mg/dL	88 (71 - 114)	84.5 (66.3 - 102.8)	95 (73 - 132.5)	84.5 (76 - 130.8)	0.125
**Repurfusion time, median (IQR)**					
Onset to repurfusion (hours)	5.9 (4.5 - 7.7)	5.5 (4.4 - 7.1)	6.5 (5.4 - 9)	5 (4.4 - 7.7)	0.138
Door to repurfusion (hours)	2.6 (2 - 3.2)	2.6 (2.2 - 3.5)	2.7 (2 - 3.2)	2.3 (1.9 - 3)	0.515
Puncture to repurfusion (hours)	0.7 (0.5 - 1.1)	0.8 (0.5 - 1.2)	0.7 (0.4 - 1.1)	0.6 (0.4 - 1.1)	0.823
**NIHSS, median (IQR)**					
ER NIHSS	17.5 (13 - 21)	17 (13 - 19)	19 (14.3 - 21)	18.5 (13 - 24.8)	0.345
Post-24h NIHSS	11 (6 - 16.3)	8 (4 - 13.8)	11 (6.3 - 16)	16.5 (8.5 - 20.5)	0.006**
NIHSS improved	5 (2.8 - 10.3)	7.5 (4.8 - 12)	4.5 (2 - 10)	5 (-1.5 - 9.3)	0.203
Vessel side, n (%)					0.830
Left	38 (55.9%)	15 (53.6%)	13 (54.2%)	10 (62.5%)	
Right	30 (44.1%)	13 (46.4%)	11 (45.8%)	6 (37.5%)	
Vessel location, n (%)					0.696
MCA	53 (77.9%)	22 (78.6%)	20 (83.3%)	11 (68.8%)	
ICA	5 (7.4%)	2 (7.1%)	2 (8.3%)	1 (6.3%)	
Combined	10 (14.7%)	4 (14.3%)	2 (8.3%)	4 (25%)	
**Pre-procedural CT, median (IQR)**					
CBF <30% of CTP	23.5 (4.5 - 43.8)	18 (0 - 46.8)	12 (0 - 28.5)	43 (32 - 51)	0.052
Tmax > 6sec of CTP	99 (71.8 - 131)	99 (75 - 138)	85 (49 - 129.5)	103 (86 - 131)	0.549
Tmax > 10sec of CTP	65 (21.8 - 81.8)	60 (30 - 90.5)	64 (15.5 - 85.5)	66 (40 - 81)	0.681
Hypoperfusion index ratio	0.6 (0.4 - 0.7)	0.5 (0.4 - 0.7)	0.6 (0.3 - 0.7)	0.6 (0.5 - 0.7)	0.600
Mismatch ration	2.9 (2.3 - 5.4)	2.5 (2.1 - 11.9)	4 (2.7 - 5.8)	2.6 (1.7 - 3.5)	0.103
Collateral score	4 (3 - 4)	4 (3.3 - 4.8)	4 (4 - 4)	3 (2 - 3)	<0.001**
NCCT-ASPECTS	9 (8 - 9)	9 (8 - 9)	8 (8 - 9)	8 (7 - 9)	0.020*
**Procedural, n (%)**					

mTICI					0.668
2B	17 (25%)	5 (17.9%)	8 (33.3%)	4 (25%)	
2C	9 (13.2%)	4 (14.3%)	2 (8.3%)	3 (18.8%)	
3	42 (61.8%)	19 (67.9%)	14 (58.3%)	9 (56.3%)	
**Post-procedural DECT, median (IQR)**					
sNCCT-ASPECTS	8 (6 - 9)	9 (7.3 - 9)	7 (6 - 8)	6.5 (4 - 8)	<0.001**
VNC-ASPECTS	6 (5 - 8)	8 (6.3 - 9)	6 (5 - 7)	5 (2 - 6)	<0.001**
IOM-ASPECTS	6 (5.5 - 7.5)	5.5 (2 - 6.8)	7 (6 - 8)	6 (4.5 - 7.5)	0.274
**Post-procedural MRI**					
Ischemic volume (mm^3) by MRI, median (IQR)	3.6 (-20.3 - 28.9)	4.9 (-13.9 - 31.8)	7.6 (-19.8 - 23.7)	-2.1 (-31.3 - 39.9)	0.734
DWI-ASPECT, median (IQR)	5 (4 - 6)	6 (5 - 8)	5 (5 - 6)	4 (2 - 5)	0.001**
**Clinical Outcomes, n (%)**					
Neurosurgical intervention	3 (4.4%)	2 (7.1%)	0 (0%)	1 (6.3%)	0.450
Inhospital length, median (IQR)	15 (9 - 19.8)	12 (8 - 15)	16.5 (11.8 - 25.8)	18 (15.3 - 21.5)	0.002**
Favorable mRS at 3-month	30 (44.1%)	16 (57.1%)	9 (37.5%)	5 (31.3%)	0.180
Excellent mRS at 3-month	16 (23.5%)	12 (42.9%)	3 (12.5%)	1 (6.3%)	0.006**
3-month mortality	6 (8.8%)	2 (7.1%)	4 (16.7%)	0 (0%)	0.180
NCCTDWI, median (IQR)	3 (2 - 4)	3 (1 - 4)	3 (2 - 3.8)	4 (3 - 5)	0.016*
VNCDWI, median (IQR)	0 (0 - 2)	1 (0 - 2.8)	0 (0 - 1)	0.5 (0 - 1.8)	0.369
Ischemic volume trend, median (IQR)	21471 (7186.5 - 46810.5)	23013 (8127 - 77734.5)	18717 (5779.5 - 46810.5)	27024 (5484 - 45735)	0.959

Data are median (IQR) or n (%). Units: Age, years; BMI, kg/m^2; Hb, g/dL; WBC, cells/μL; Platelet, ×10^3/μL; Cholesterol/LDL, mg/dL; time intervals (onset-to-, door-to-, puncture-to-reperfusion), hours; in-hospital length, days; CTP volumes (CBF <30%, Tmax >6 s, Tmax >10 s), mL; MRI ischemic volume, mL. Scores are unitless unless otherwise noted: NIHSS (points), mRS, collateral score (0–5), ASPECTS (0–10), mTICI (2B–3). ASPECTS differences (NCCTDWI, VNCDWI) are in points; ischemic volume trend is in mL. Tests: Mann–Whitney U or

Kruskal–Wallis for continuous variables; chi-square or Fisher (or Fisher–Freeman–Halton) for categorical variables. Significance: *p*<0.05; **p**<0.01.
